# Therapeutic Effects of Methanol Extract from *Euphorbia kansui* Radix on Imiquimod-Induced Psoriasis

**DOI:** 10.1155/2017/7052560

**Published:** 2017-07-02

**Authors:** Soo Jeong Kim, Ye Won Jang, Kyung Eun Hyung, Da Kyoung Lee, Kee Hyeob Hyun, So-Young Park, Eon-Sub Park, Kwang Woo Hwang

**Affiliations:** ^1^College of Pharmacy, Chung-Ang University, 84 Heukseok-ro, Dongjak-gu, Seoul 06974, Republic of Korea; ^2^College of Pharmacy, Dankook University, 119 Dandae-ro, Dongnam-gu, Cheonan-si, Chungnam 31116, Republic of Korea; ^3^College of Medicine, Chung-Ang University, 84 Heukseok-ro, Dongjak-gu, Seoul 06974, Republic of Korea

## Abstract

The roots of *Euphorbia kansui*, which belong to the family Euphorbiaceae, have been used as a traditional medicine for the treatment of various diseases such as diabetes, ascites, and leukemia. Recently, it was reported that the methylene chloride fraction of *E. kansui* radix (EKC) regulated the differentiation of Th17 cells and alleviated the symptoms of Th17-related inflammatory bowel disease. Imiquimod (IMQ), a TLR7/8 agonist, has been used to induce psoriasis in a mouse model. In this study, we evaluated the effect of EKC in an IMQ-induced psoriasis model. EKC effectively inhibited the production of interleukin-17A and interferon-*γ* in vitro. On this basis, EKC was administered to an animal model of psoriasis. Acanthosis and the infiltration of inflammatory cells into the dermis were significantly reduced by EKC. EKC also inhibited the expression of IL-17A, IL-22, IL-23, IL-12, and RAR-related orphan receptor gamma t (ROR*γ*t) in the spleen, skin-draining lymph nodes, and the skin. Additionally, EKC inhibited the activity of dendritic cells but not that of keratinocytes. In conclusion, EKC ameliorated the symptoms of psoriasis through inhibition of Th17 differentiation and activation of dendritic cells. These effects are expected to be beneficial in the treatment and prevention of psoriasis.

## 1. Introduction

Psoriasis is recognized as the most common type of chronic inflammatory dermatosis, which is caused by a disorder of the immune system in which T cells play a primary role [1]. Psoriatic inflammation was initially considered to be mediated by T-helper 1 (Th1) cells that produced interferon-*γ* (IFN-*γ*) [2]. However, research increasingly indicates that T-helper 17 (Th17) cells, which produce interleukin-17 (IL-17) and interleukin-22 (IL-22), are critical in the pathogenesis of psoriasis [3, 4]. Th17 cells are important in the host defense against specific extracellular bacteria, and they have been associated with various autoimmune diseases, including rheumatoid arthritis, inflammatory bowel disease, and multiple sclerosis [5–8]. In the skin, after plasmacytoid dendritic cells (pDCs) in the epidermis have been activated by a trigger (stress, microorganisms, or DNA mutation), they secrete a high level of IFN-*α*, which activates dermal DC and initiates T cell-mediated immunity. Activated dendritic cells produce IL-12 and IL-23, which induce the differentiation of naive T cells into Th17 cells. The cytokines and activated Th17 cells advance the pathogenesis of psoriasis [9]. The pathogenic symptoms of psoriasis are characterized by hyperplasia of epidermal keratinocytes, scaling, and infiltration of neutrophils and lymphocytes [10]. Many immune-derived cytokines, including IL-23, IL-17A, IL-22, TNF-*α*, and IFN-*γ*, are involved in disease development [11, 12]. Various studies of patients with psoriasis have demonstrated that the amelioration of psoriasis was associated with reduced Th17 response. It was reported that ustekinumab, a monoclonal antibody (mAb) to IL-12/23 p40, significantly reduced IL-23p19 and IL-12/23p40 gene expression compared with baseline levels in the lesions of psoriatic patients [13, 14]. In addition, secukinumab and ixekizumab, specific mAbs for human IL-17A, are in phase III clinical development for the treatment of plaque psoriasis [15–19].

Imiquimod (IMQ) has been approved for the treatment of genital warts and actinic keratosis but occasionally leads to the development of psoriasis in humans [20–24]. IMQ is a potent agonist of TLR7/8 and facilitates local and acquired immune responses [25]. Experimental data showed that IMQ-induced dermatitis in mice closely resembled human psoriatic lesions, not only in the histological characteristics but also in the development of lesions [26]. In addition, IMQ application induced the epidermal expression of IL-23, IL-17A, and IL-17F, as well as increased their expression in splenic Th17 cells [27–29]. This experimental method has therefore allowed the elucidation of new therapies for psoriasis. Recent studies have established the mechanism of pathogenesis and the molecular targets for the treatment of psoriasis through an IMQ-induced mouse model [30–32].

The roots of *Euphorbia kansui* (Kansu), which are plants belonging to the Euphorbiaceae family, have been used as a traditional medicine throughout the Far East. Many herbs in this family have been traditionally utilized to treat various diseases such as edema, ascites, and asthma. Approximately 100 chemical compounds have been isolated and identified from *E. kansui.* Diterpenoids (e.g., kansuinine A and B) and triterpenoids (e.g., euphol) are the main chemical constituents responsible for the biological effects, which include antiviral, antiproliferative, and immunomodulatory activities [33]. The ethanol extract of *E. kansui* has the ability to activate lymphocytes, which enhanced its capacity to remove virus-infected cells [34]. Terpenoid compounds derived from the roots of *E. kansui* showed a significant inhibition of proliferative activity of embryonic cells and intestinal epithelioid cells [35, 36]. It was reported that ingenane-type diterpenes from Kansu modulated IFN-*γ* production by regulating nuclear factor kappa-light-chain-enhancer of activated B cells (NF-*κ*B) [37].

Th17 cell activity was reduced by the activation of extracellular signal-regulated protein kinase (ERK) and increased by the phosphorylation of Stat3. A topical application of euphol, a triterpenoid isolated from the roots of *E. kansui,* significantly inhibited TPA-induced ear edema through activation of ERK [38]. Moreover, kansuinine A and B, chemical compounds from *E. kansui*, have an inhibitory effect on IL-6-induced Stat3 activation [39]. Previous studies have identified that ethanol extracts of *E. kansui* radix inhibited Th17 cell differentiation and increased the division of regulatory T cells (Treg cells) in Th17-driving conditions (unpublished data). In addition, the methylene chloride fraction of *E. kansui* radix extracted with methanol (EKC) repressed DSS-induced colitis (unpublished data). These regulatory effects on Th17 and related factors led us to investigate the in vivo effect of Kansu extract on the development of psoriasis.

This study was designed to investigate the effect of EKC in an IMQ-induced psoriasis model and to explore underlying mechanism on its therapeutic efficacy.

## 2. Materials and Methods

### 2.1. Mice

Female Balb/c mice were purchased from Orient Bio (Sungnam, Korea). The mice were aged 6–9 weeks for all experiments. Animals were maintained in a specific pathogen-free environment under the following controlled conditions: temperature, 21 ± 3°C; relative humidity, 50 ± 10%; and illumination, 10 h light and 14 h darkness. All studies and procedures were conducted in accordance with the National Institutes of Health Guide for the care and use of laboratory animals, and the protocol was approved by the Institutional Animal Care and Use Committee of the Laboratory Animal Research Center of Chung-Ang University.

### 2.2. Materials

The materials used in this study included Aldara cream (5% imiquimod, Dong-Ah Pharmaceutical, Seoul, Korea) and human IL-17A recombinant protein (ebioscience, San Diego, USA). The roots of *E. kansui* were obtained from a commercial herbal drug market (Jaesung Medicinal Herbal Drug Market, Seoul, Korea). The voucher specimens were stored at the Pharmacognosy Laboratory of the College of Pharmacy, Dankook University, Korea. The dried roots of *E. kansui* were pulverized and extracted with 90% aqueous methanol. The methanolic extract was dissolved in water and partitioned with *n*-hexane and methylene chloride. The methylene chloride fraction of the methanol extract from *E. kansui* radix was referred to by the abbreviation EKC.

### 2.3. Purification of CD4^+^ T Cells and Th17/Th1 Differentiation

The spleens were extracted from Balb/c mice and naive CD4^+^ T cells were purified by using a magnetic cell sorting system (MACS® separation, Miltenyi Biotech, Bergisch Gladbach, Germany). The cells were cultured in RPMI 1640 culture media with 10% heat-inactivated FBS (Cellgro, Herndon, VA, USA), 100 U/mL penicillin (Cellgro), 0.1 mg/mL streptomycin (Cellgro), 2 mM L-glutamine (Cellgro), and 0.05 *μ*M 2-mercaptoethanol (Sigma-Aldrich, St. Louis, USA) at 37°C in a 5% CO_2_-humidified incubator. Plate-bound anti-CD3 antibody (1 *μ*g/mL, ebioscience) and anti-CD28 antibody (1 *μ*g/mL, ebioscience) were used to stimulate T cells. Recombinant mouse IL-6 (25 ng/mL, BD, San Jose, CA, USA) and TGF-*β* (2.5 ng/mL, BD) were used for Th17 differentiation, and IL-2 (10 ng/mL, BD), IL-12 (5 ng/mL, Biosource, Camerillo, CA, USA), and anti-IL-4 (5 *μ*g/mL) were added to induce differentiation into Th1 cells.

### 2.4. Induction or Evaluation of Imiquimod-Induced Psoriasis

**Figure 1:**
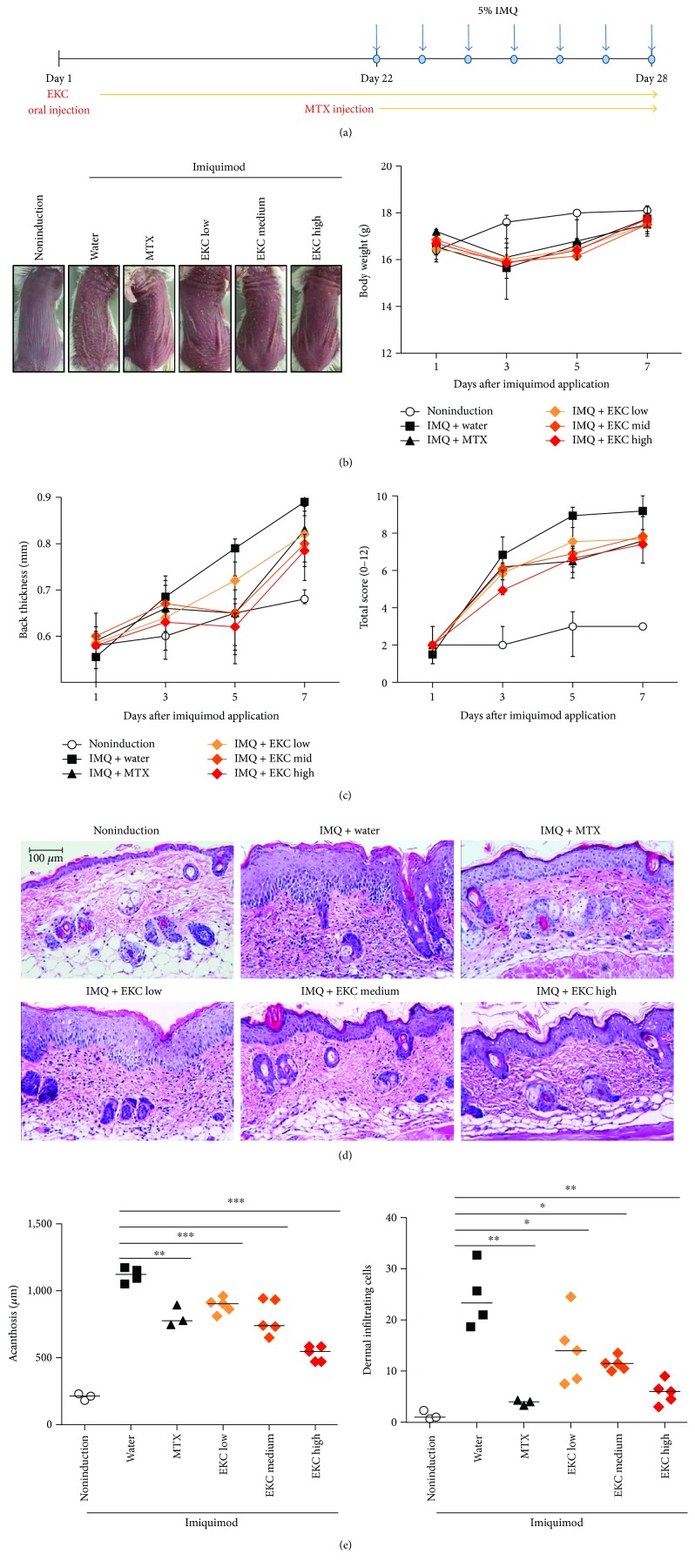
Experimental procedure and effects of the oral administration of EKC on dermatitis lesions. (a) Experimental overview. Day 1 was defined as the first administration of EKC (20 mg/kg, 100 mg/kg, and 200 mg/kg). After 22 days, imiquimod cream was applied to the shaved back skin of the mice. Methotrexate (MTX) was used as positive control. (b) Imiquimod was applied daily to Balb/c mice. After 7 days, pictures of mice were taken and the phenotypical symptoms of the mouse back skin were observed. The variation in body weight was measured for 7 days. (c) Dermatitis scores (back thickness, redness, and scaling) were evaluated every other day from day 22 to day 28. Back thickness and total score (erythema plus thickness plus scaling) are presented as mean ± SD (*n* = 5). (d) H&E staining of skin tissue treated with MTX or different doses of EKC (original magnification, ×200). (e) Acanthosis was evaluated by measuring the length of the epidermal cell layers. Three sections per mouse sample were analyzed. The dermal infiltrating cells were counted from three random sections of each sample. Bars represent the median. ^∗^*p* < 0.05; ^∗∗^*p* < 0.01; and ^∗∗∗^*p* < 0.001 compared with imiquimod + water group.

Balb/c mice were separated into six groups each containing five animals. To induce skin inflammation in the mice using the inflammation response modifier drug imiquimod (IMQ), five of the groups received a consecutive daily topical dose (62.5 mg) of commercially available 5% imiquimod cream (3.215 mg of active compound) on shaved back skin for 7 days. The noninduced control group was treated similarly with a vehicle cream (Vaseline, Unilever, London, UK). For oral administration, EKC was dissolved in Dimethylacetamide (DMA) and diluted in tap water to achieve a final DMA concentration < 5%. EKC was daily administered from 22 days before IMQ application to the final of the study, and methotrexate was used as a positive control (Figure 1(a)). The psoriasis-induced groups were IMQ + water, IMQ + MTX (1 mg/kg methotrexate; Yuhan corporation, Seoul, Korea), and IMQ + EKC low (20 mg/kg), middle (100 mg/kg), and high (200 mg/kg). Back thickness was measured using digital thickness gauge (Bluebird, Seoul, Korea). A scoring system based on the clinical Psoriasis Area and Severity Index (PASI) was used to score the severity of the skin inflammation. Erythema, thickness, and scaling were scored independently on a scale from 0 to 4 as follows: 0, none; 1, slight; 2, moderate; 3, marked; and 4, very marked. The cumulative score served as a measure of the severity of inflammation (scale 0–12).

### 2.5. Histological Analysis

For the histological examinations, a skin sample with a diameter of 3 mm was removed from the back skin of psoriasis-induced mice on the final day of the administration schedule and fixed in 10% phosphate-buffered formalin (pH 7.2). The biopsies were embedded in paraffin, cut, and stained with hematoxylin and eosin for the evaluation of acanthosis and dermal infiltrating cells. The staining was analyzed by using a microscope and observing three sections from each mouse.

### 2.6. Cell Culture

JAWSII cells, a mouse dendritic cell line, were cultured in RPMI 1640 medium (Cellgro) supplemented with FBS (10%, heat-inactivated; Cellgro), penicillin (100 U/mL; Cellgro), streptomycin (0.1 mg/mL; Cellgro), L-glutamine (2 mM; Cellgro), 2-mercaptoethanol (0.05 *μ*M; Sigma), and mouse granulocyte-macrophage colony-stimulating factor (mGM-CSF; 5 ng/mL, R&D Systems, Minneapolis, MN, USA) at 37°C in a 5% CO_2_-humidified incubator. The cells were seeded at 1 × 10^6^ cells/well in a 24-well plate and stimulated for 24 h with conditioned medium containing 1 *μ*g/mL imiquimod, with or without EKC (5 or 10 *μ*g/mL). The cell supernatants were collected to measure IL-12p40 and IL-23p19. HaCaT human keratinocyte cell lines were cultured in DMEM (Cellgro) supplemented with 10% heat-inactivated FBS, penicillin, and streptomycin at 37°C in a 5% CO_2_-humidified incubator. The cells were seeded at 1 × 10^6^ cells/well in 24-well plates and treated with human IL-17A (100 ng/mL, ebioscience) and EKC (5 or 10 *μ*g/mL) for 24 h. After treatment, the cells were harvested for RNA extraction.

### 2.7. Sample Preparation for ELISA

CD4^+^ T cells (1 × 10^6^) were plated in 24-well plates in Th17- and Th1-driving conditions and treated with 5 or 10 *μ*g/mL EKC. The supernatants were harvested at days 1, 2, and 3. The concentration of IL-17A, TNF-*α*, IFN-*γ*, and IL-12p40 in each sample was detected by ELISA. Splenocytes, axillary lymph node (ALN) cells, and brachial lymph node (BLN) cells from mice were plated on flat-bottom 24-well plates (1 × 10^6^ cells/well) in the presence of plate-bound anti-CD3 Ab, and the supernatant was collected after 24 h and 48 h. The concentration of IL-17A, IL-12p40, TNF-*α*, and IFN-*γ* in each sample was detected by ELISA using Ab pairs. Skin biopsies from back skin were collected at the end of the experimental day. The samples were homogenized and extracted using T-PER tissue protein extraction reagent (Thermo Fisher Scientific, San Jose, CA, USA) in the presence of a protease inhibitor cocktail (Thermo Fisher Scientific) and phosphatase inhibitor cocktail (Thermo Fisher Scientific). The protein extracts were centrifuged at 10,000*g* for 5 min. The protein concentration of the skin extracts was estimated and quantified using BCA protein assay reagents (Thermo Fisher Scientific). For the measurement of total serum IgG1 and cytokines, blood specimens were obtained from the retro-orbital sinus on day 28. The serum was separated and stored at −80°C until use.

### 2.8. RNA Isolation and Real-Time RT-PCR

Splenocytes, ALN cells, and BLN cells (1 × 10^6^) from mice were activated ex vivo by incubation with anti-CD3 (1 *μ*g/mL, BD) for 2 days, and total RNA was isolated from each sample using TRIzol (Invitrogen, Carlsbad, CA, USA). Skin samples were homogenized and centrifuged at 12,000*g* for 10 min, and the total RNA was extracted from the biopsies of the back skin using TRIzol reagent. RNA was transcribed to cDNA at 42°C for 1 h in a total reaction volume of 25 *μ*L, which contained 5× RT buffer, 10 mM dNTPs (200 units), MMLV-RT (Moloney murine leukemia virus reverse transcriptase), and 100 pmol oligo-dT primer. The cDNA was then used for quantitative real-time PCR with 2× iQTM SYBR Green Supermix (Bio-Rad, Hercules, CA, USA) to determine the mRNA levels of IL-17A, IL-22, IL-12p40, IL-23p19, ROR*γ*t, human IL-8, human IL-36*γ*, human CCL20, and GAPDH. To confirm PCR specificity, the PCR products were subjected to melting curve analysis. The comparative threshold method was used to calculate the relative amount of mRNA in the experimental samples compared with the control samples. Gene expression was normalized to the expression of GAPDH.

### 2.9. Flow Cytometry Analysis

Splenocytes were collected in FACS buffer and stained with the following antibodies: Thy1.2 FITC, CD4 APC, CD8 APC, *γδ* TCR PE-Cyanine5, CD19 PE, CD11b FITC, F4/80 PE, and CD11c FITC (BD and ebioscience). The stained cells were analyzed using a FACSCalibur flow cytometer and Cell Quest analysis software (BD Bioscience).

### 2.10. Western Blot

Skin samples from the back lesions of mice were cut into pieces, snap-frozen in liquid nitrogen, and stored at −80°C until use. The samples were homogenized in T-PER buffer (Thermo Fisher Scientific) in the presence of a protease inhibitor cocktail and a phosphatase inhibitor cocktail. Equal amounts of proteins were separated by SDS-PAGE and transferred to PVDF (polyvinylidene fluoride) membranes. The blots were blocked and incubated at 4°C overnight with antibodies against p-I*κ*B and *β*-actin (Cell signaling, Beverly, MA, USA). The membranes were then incubated with anti-rabbit IgG or anti-mouse IgG HRP-linked antibodies (cell signaling). Chemiluminescence was measured by using the chemiDoc system (Bio-rad) and analyzed with Quantity One software (Bio-Rad).

### 2.11. Cell Viability Assay

JAWSII cells (2 × 10^5^) were plated in a 96-well multiplate, EKC was added, and the cells were cultured for 24 h. After culture, 10 *μ*L MTT [3-(4, 5-dimethylthiazolyl-2)-2, 5-diphenyltetrazolium bromide] solution (5 mg/mL, Sigma-Aldrich) was added to each well and incubated at 37°C for 2 h. One hundred microliters of solubilization solution (0.04 N HCl in isopropanol) was added to each well. The plate was evaluated at 570 nm wavelength using a microplate reader (Emax, Molecular Devices, Sunnyvale, CA, USA).

### 2.12. Statistical Analysis

Data were expressed as the mean ± SD, and statistical significance was analyzed by using Student's *t*-test. Different levels of statistical significance were denoted as ^∗^*p* < 0.05, ^∗∗^*p* < 0.01, and ^∗∗∗^*p* < 0.001.

## 3. Results

### 3.1. Methylene Chloride Fraction of *E. kansui* Radix Suppresses Th17-Specific Cytokines

**Figure 2:**
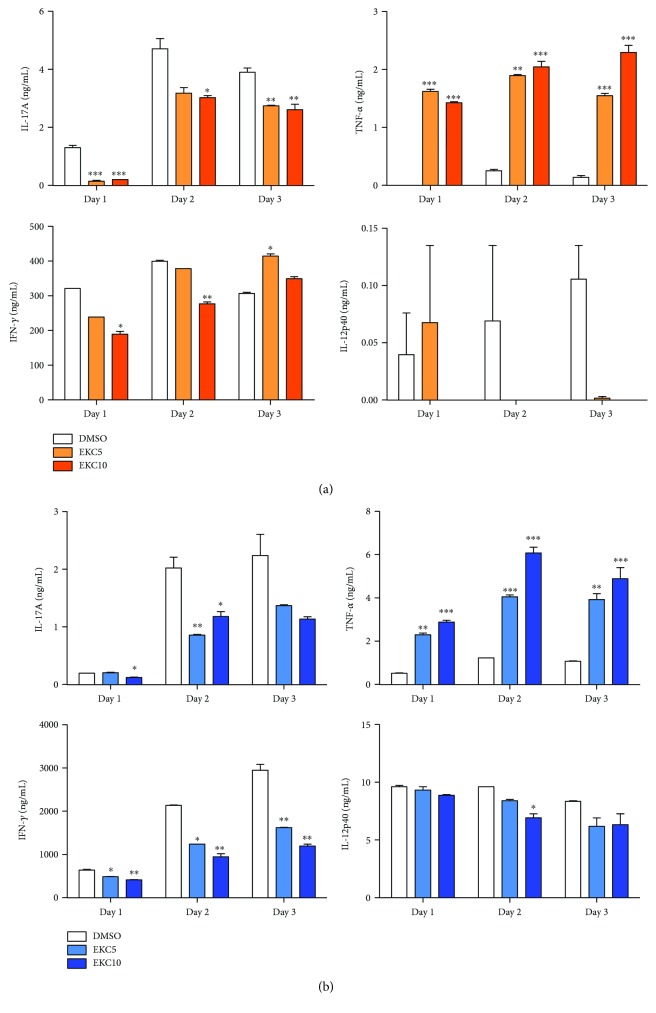
Decreased secretion of IL-17A and IFN-*γ* in both Th17- and Th1-driving conditions by EKC. (a) CD4^+^ T cells were purified from Balb/c mice and activated with 1 *μ*g/mL of plate-bound anti-CD3/CD28 antibodies in the presence of 25 ng/mL IL-6 and 2.5 ng/mL TGF-*β*, with or without EKC. Production of IL-17A, IFN-*γ*, TNF-*α*, and IL-12p40 was detected by ELISA. (b) Isolated CD4^+^ T cells were stimulated with plate-bound anti-CD3/CD28 antibodies with or without *E. kansui* under Th1-driving cytokine conditions (10 ng/mL IL-2, 5 ng/mL IL-12, and 5 *μ*g/mL anti-IL-4) for 3 days. The concentration of cytokines in the supernatant was detected by ELISA. Values represent the mean ± SD. (^∗^*p* < 0.05; ^∗∗^*p* < 0.01; and ^∗∗∗^*p* < 0.001 compared with the DMSO-treated group).

To determine the effect of EKC on Th17 and Th1 polarization, CD4^+^ T cells were treated with EKC under Th17- and Th1-driving conditions. After 3 days, the supernatant was harvested and analyzed by ELISA. In Th17-driving conditions, the production of IL-17A was significantly decreased and IFN-*γ* secretion was slightly decreased by EKC treatment. In contrast to the results for IL-17A and IFN-*γ* secretion, TNF-*α* secretion significantly increased (Figure 2(a)). In Th1-driving conditions, the EKC-treated sample showed consistent results with Th17-driving conditions (Figure 2(b)). IL-12p40 was not detected in Th17-driving conditions, but a decreasing trend was observed in the presence of EKC in Th1-driving conditions (Figure 2(b)).

### 3.2. EKC Prevents Epidermal Hyperplasia and Infiltration of Inflammatory Cells in IMQ-Induced Psoriasis

In the pathogenesis of psoriasis, the immune response of Th17 cells is recognized as a critical factor. To investigate whether there was a beneficial effect on psoriasis as well, EKC was orally administered to mouse models. EKC was administered daily for 3 weeks at three escalating doses (20, 100, and 200 mg/kg). After 21 days, the animals were challenged topically on the back skin with IMQ according to the schedule summarized in Figure 1(a). The phenotype of psoriasis (back thickness, redness, and scaling) was observed throughout the 7-day period of IMQ application. Mice that were orally EKC displayed reduced thickness at day 5, less culminated redness at day 3, and sparser scales (data not shown). The total scores for all groups in the experiment are depicted in Figure 1(b). Significant difference in the disease severity was observed in EKC and MTX groups compared with IMQ + water group (Figure 1(b) and Table 1). The body weight of the mice that received 20, 100, and 200 mg/kg EKC did not differ significantly from that of the noninduced control group (Figure 1(c)). Similar to the clinical score, the histological analyses of skin samples at day 7 of IMQ application revealed that EKC treatment alleviated acanthosis and infiltration of inflammatory cells in a dose-dependent manner. MTX decreased this psoriatic symptoms (Figures 1(d) and 1(e)). Thus, MTX treatment and EKC administration ameliorated dermatitis in IMQ-induced skin in both clinical and pathological measures.

### 3.3. IMQ-Induced Splenomegaly Is Slightly Decreased by EKC Treatment, and the Population of T Cells in the Spleen Is Not Affected by EKC

**Figure 3:**
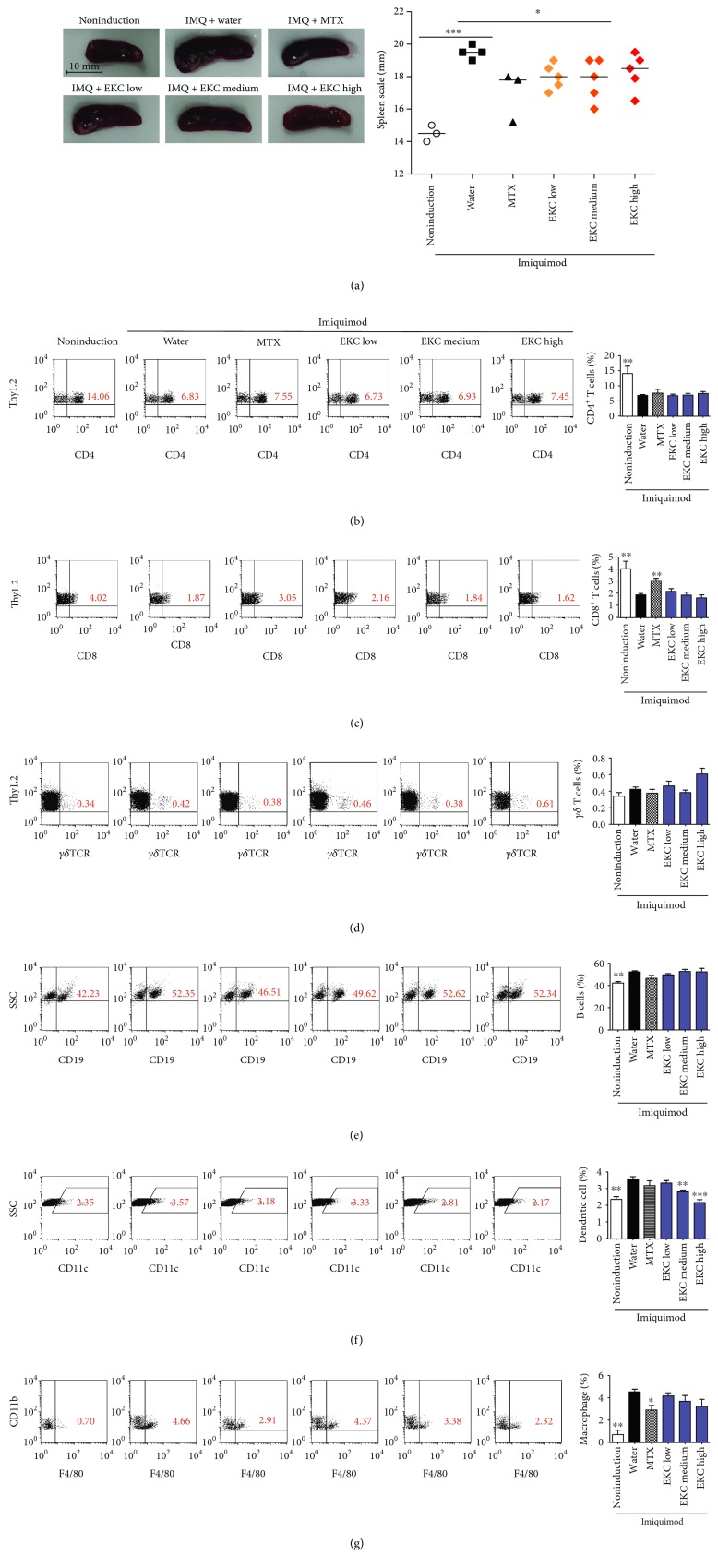
Effects of EKC on the spleen and analysis of cellular composition. (a) Photographs of the spleen on day 28. Each spleen was measured, and the data presented are the median (*n* = 5). (b–g) Mice were treated with IMQ or vehicle cream for 7 days consecutively and sacrificed. Splenocytes were analyzed for the percentage of T cells ((b) Thy1.2^+^CD4^+^; (c) Thy1.2^+^CD8^+^; (d) Th1.2^+^*γδ* TCR^+^; (e) B cells [CD19^+^]; (f) dendritic cells [CD11c^+^]; and (g) macrophages [F4/80^+^CD11b^+^]) by flow cytometry. Numbers indicate the mean percentage of cells present within a quadrant or gate (*n* = 5 mice/group). Graphs are presented as the mean ± SD (*n* = 5). ^∗^*p* < 0.05; ^∗∗^*p* < 0.01; and ^∗∗∗^*p* < 0.001 compared with imiquimod + water group.

IMQ treatment resulted in a significant enlargement of the spleen (Figure 3(a)), and the number of cells increased in dermatitis-induced groups compared to that in the noninduced control (data not shown). The administration of low and medium concentrations of EKC and MTX slightly suppressed the observed increase (Figure 3(a)). In addition, the cellular composition of the spleen was determined by flow cytometry. IMQ treatment induced a decrease in the percentage of CD4^+^ and CD8^+^ T cells. However, no obvious differences were observed in the EKC treatment groups compared to those reported with IMQ. However, CD8^+^ T cells were slightly restored by MTX application (Figures 3(b) and 3(c)). The percentage of *γδ* T cells somewhat increased in IMQ-treated mice, while administration of EKC and MTX did not change this percentage (Figure 3(d)). In contrast to that of T cells, the percentage of B cells, dendritic cells, and macrophages significantly increased in mice after the topical treatment with IMQ. In particular, the IMQ-induced increase in dendritic cell population decreased when EKC was administered at medium and high concentrations. On the contrary, the elevated population of macrophage was reduced by MTX, whereas the effect of EKC was insignificant (Figures 3(e), 3(f), and 3(g)). Overall, IMQ caused systemic effects on the cellular composition of the spleen. EKC was likely to regulate the population of dendritic cells altered by IMQ-induced psoriasis, with a tendency in regulating that of macrophages but not that of T cells and B cells.

### 3.4. EKC Inhibits Th17 Cell Differentiation in the Lymphoid Organs of the Psoriatic Model

**Figure 4:**
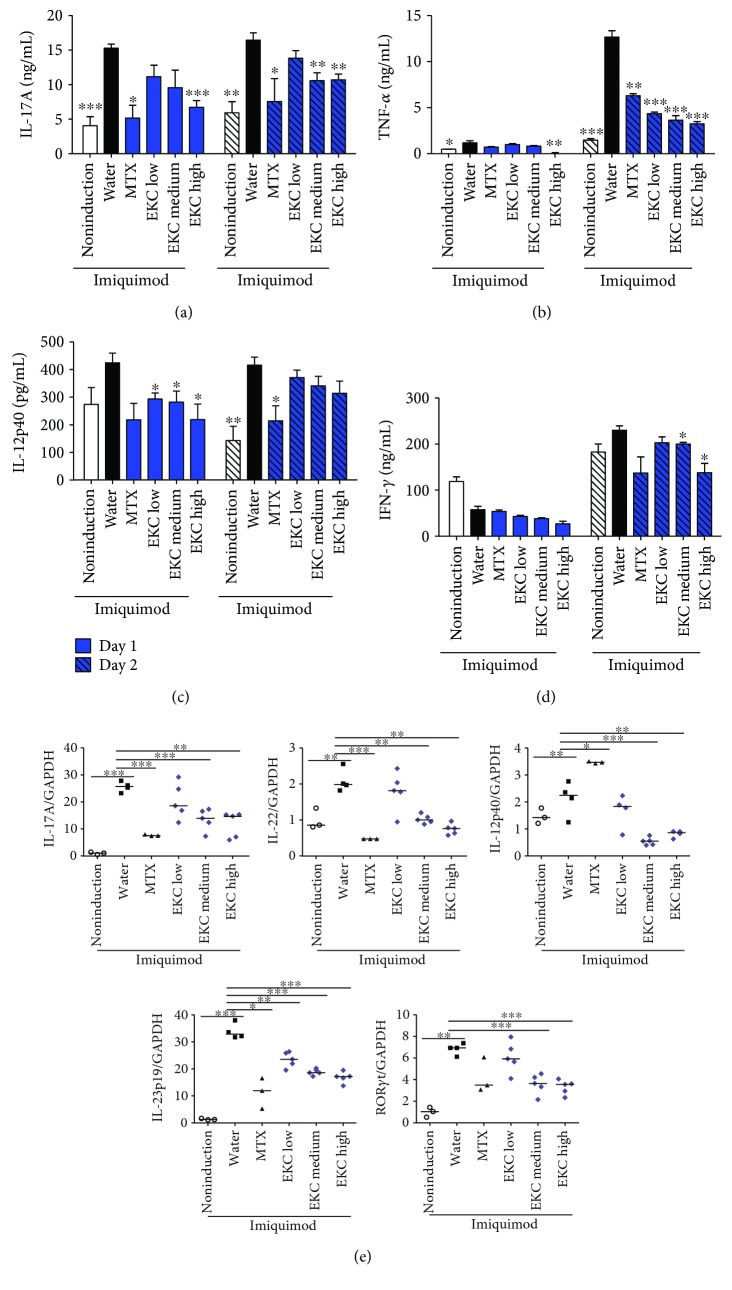
Effects of oral administration of EKC in splenocytes. (a–d) Spleen cells were purified from mice on day 28, stimulated with anti-CD3 Ab for 24 h and 48 h, and the cytokine levels were measured by ELISA. Values represent the mean ± SD. (e) Spleen cells were purified from mice on day 28 and stimulated with anti-CD3 Ab for 2 days. Total mRNA was extracted, and the levels of IL-17A, IL-22, IL-23p19, IL-12p40, and RORγt were evaluated by real-time PCR. Values represent the median. ^∗^*p* < 0.05; ^∗∗^*p* < 0.01; and ^∗∗∗^*p* < 0.001 compared with the imiquimod + water group.

To determine the functionality of the spleen cells, splenocytes were activated ex vivo by anti-CD3 for 2 days and Th17-associated factors were analyzed. First, the supernatant was collected and cytokine levels were detected. IMQ treatment remarkably enhanced the production of IL-17A and TNF-*α*, which is the signature of Th17 cytokines; however, administration of medium and high concentrations of EKC repressed this increase on days 1 and 2 (Figures 4(a) and 4(b)). On day 2, secretion of Th1 cytokines, such as IL-12p40 and IFN-*γ*, was higher than that in the vehicle cream application group (Figures 4(c) and 4(d)), whereas EKC significantly suppressed the release of IL-12p40 (day 1) and IFN-*γ* (day 2) (Figures 4(c) and 4(d)). The intake of the positive control of (MTX) or EKC decreased the production of the two Th17 (IL-17A and TNF-*α*) and two Th1 (IL-12p40 and IFN-*γ*) cytokines (Figures 4(a), 4(b), 4(c), and 4(d)). To investigate the alteration of molecular levels, total RNA was extracted from anti-CD3-stimulated splenocytes and the gene levels were analyzed using real-time PCR. Similar to the results reported for protein secretion, an increase in mRNA levels of genes related to Th17 and Th1 responses in psoriasis IL-17A, IL-22, IL-23p19, ROR*γ*t, and IL-12p40 was observed in IMQ-treated group (Figure 4(e)). At three analyzed concentrations of EKC and MTX groups, the expression levels of the Th17- and Th1-related cytokine genes were efficiently downregulated (Figure 4(e)). Moreover, the gene level of a Th17-specific transcription factor, ROR*γ*t, was diminished by EKC administration (Figure 4(e)).

It is known that a lymphoid organ near the local inflammatory sites generally participates in antigen presentation, lymphocyte differentiation, and proliferation, to accomplish the rapid and effective elimination of antigen. For these reasons, the effects of EKC were examined on axillary lymph nodes and brachial lymph nodes during IMQ-induced skin inflammation.

**Figure 5:**
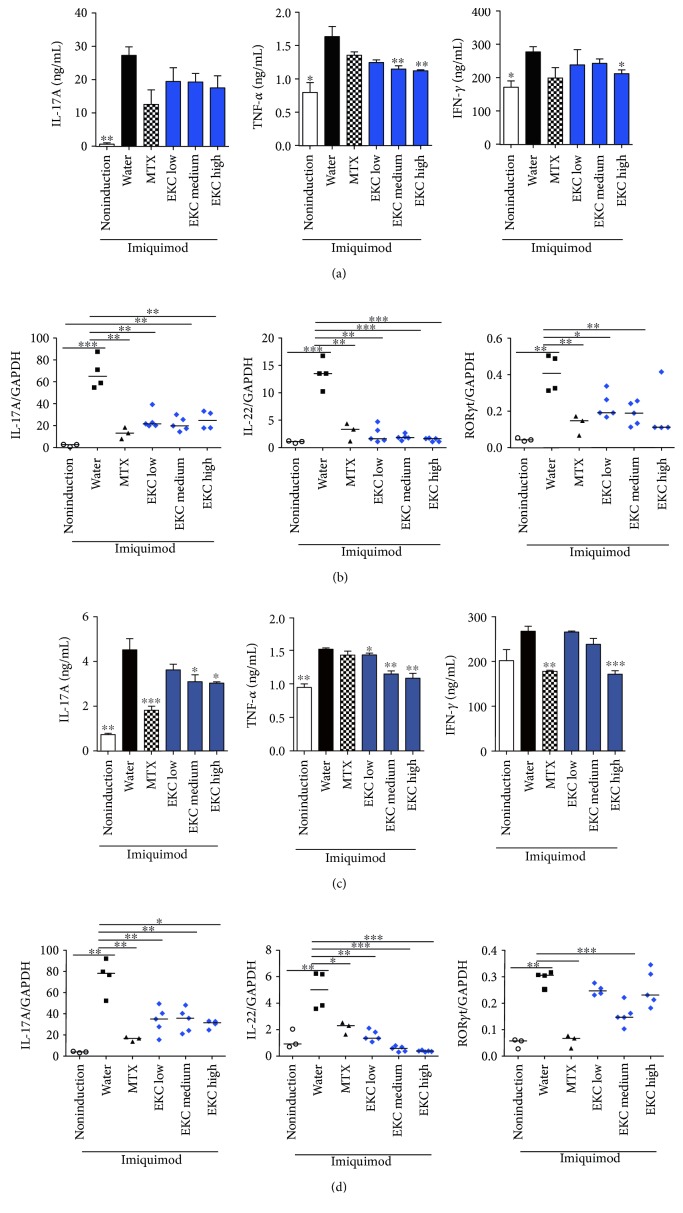
Effects of oral administration of EKC in axillary lymph nodes and brachial lymph nodes of psoriasis-induced mice. (a) At the end of the experimental day, mice were sacrificed and the axillary lymph node was isolated. The axillary lymph node cells were stimulated with anti-CD3 Ab for 2 days, and the supernatant was harvested. The cytokine levels were measured by ELISA. Values represent the mean ± SD. (b) The axillary lymph node cells were stimulated with plate-bound anti-CD3 Ab for 2 days. Total mRNA was extracted, and the levels of IL-17A, IL-22, and RORγt were measured by real-time PCR. Values represent the median. (c) At the end of the experimental day, mice were sacrificed and the axillary lymph node was isolated. The axillary lymph node cells were stimulated with anti-CD3 Ab for 2 days, and the supernatant was harvested. Cytokine levels were measured by ELISA. (d) The brachial lymph node cells were stimulated with plate-bound anti-CD3 Ab for 2 days. Total mRNA was extracted, and the levels of IL-17A, IL-22, and RORγt were measured by real-time PCR. Values represent the median. ^∗^*p* < 0.05; ^∗∗^*p* < 0.01; and ^∗∗∗^*p* < 0.001 compared with the imiquimod + water group.

In the axillary lymph node, the increase in inflammatory cytokines, especially TNF-*α* and IFN-*γ*, was suppressed by oral administration of EKC (Figure 5(a)). The expression levels of Th17-associated genes were significantly inhibited by EKC administration (Figure 5(b)). After EKC treatment, the production of IL-17A, TNF-*α*, and IFN-*γ* in brachial lymph node cells was also lower than that in the IMQ-only treatment group (Figure 5(c)). As expected, IL-17A, IL-22, and ROR*γ*t levels were also depressed by EKC (Figure 5(d)). Thus, these results showed that EKC negatively regulated Th17 differentiation in the IMQ-induced psoriasis model.

### 3.5. EKC Lowers Elevated Inflammatory Cytokines in Skin Lesion and Alleviates Systemic Immune Activation

**Figure 6:**
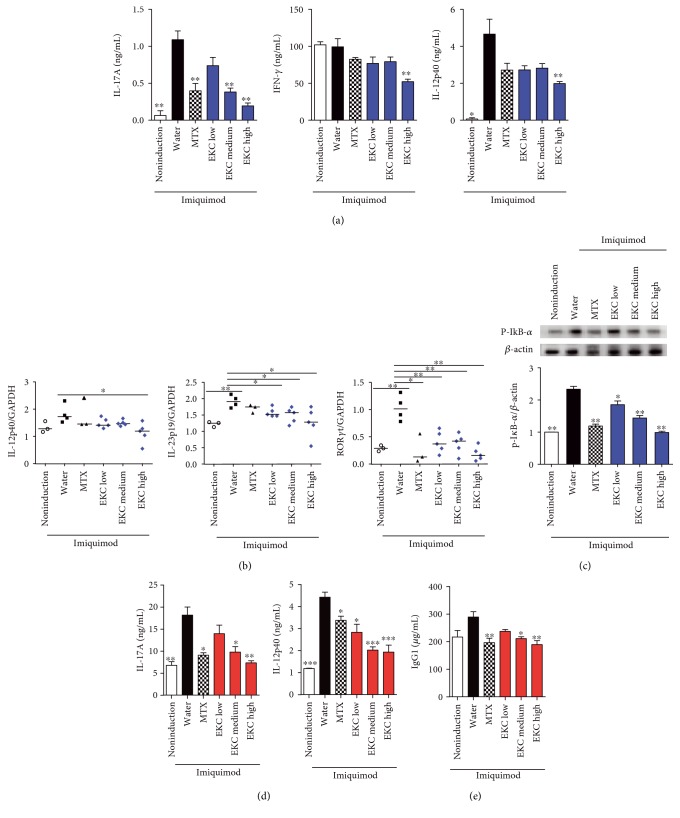
Inhibition of Th17 associated factors in inflammatory skin lesions and serum by EKC. (a) Mice were sacrificed, and protein was extracted from the back skin of each mouse. Cytokine levels were measured by ELISA. Values represent the mean ± SD. (b) Total mRNA was extracted from the skin using TRIzol, and the levels of IL-23p19 and IL-12p40 were measured by real-time PCR. Values represent the median. (c) The skin samples were prepared by homogenization with T-PER protein extraction buffer. p-I*κ*B-*α* expression levels were determined by Western blot analysis. The intensity of β-actin staining in each sample was used as a loading control. (d) The concentration of IL-17A and IL-12p40 collected in serum on day 28 was determined by ELISA. Values represent the mean ± SD. (e) Concentration of IgG1 in the collected serum was measured by ELISA. Values represent the mean ± SD. ^∗^*p* < 0.05; ^∗∗^*p* < 0.01; and ^∗∗∗^*p* < 0.001 compared with the IMQ + water group.

To clarify whether EKC effectively suppressed the function of Th17 cell in skin lesion, similar to the effects observed in lymphoid organs, Th17-related factors were analyzed in IMQ-treated skin. Compared with the noninduced control group, the production of IL-17A and IL-12p40 was exceptionally increased in skin samples from IMQ-treated mice. In contrast, IL-17A was significantly decreased in MTX and EKC groups. The high concentration of EKC group displayed lower IFN-*γ* and IL-12p40 production than the IMQ + water group did (Figure 6(a)). Additionally, the cytokine genes supporting Th17 and Th1 differentiation, IL-23p19 and IL-12p40, were significantly diminished, and the Th17 signature transcription factor, ROR*γ*t, was downregulated by EKC (Figure 6(b)).

Increased levels of IL-17A in the skin induce phosphorylation of I*κ*B in keratinocytes, which activates NF-*κ*B signaling. Therefore, the expression levels of p-I*κ*B-*α* were evaluated in a Western blot of skin tissue. MTX treatment decreased expression of p-I*κ*B. In the EKC-treated group, the phosphorylation of I*κ*B was impeded in a dose-dependent manner (Figure 6(c)).

In addition, serum cytokine levels were detected by ELISA. IMQ induced elevation of IL-17A and IL-12p40, whereas MTX and EKC inhibited these cytokine secretions in a dose-dependent manner (Figure 6(d)). As an expanded population of B cells was observed in the IMQ-treated group (Figure 3(e)), the IgG level in the serum was measured. As shown in Figure 6(e), total IgG1 levels in IMQ-treated mice were higher than those in the noninduced control group (*p* > 0.05), whereas MTX application and EKC administration significantly decreased the IgG1 levels in a dose-dependent manner.

### 3.6. EKC Regulates the Activity of Dendritic Cells and Does Not Affect Keratinocyte

To verify whether EKC affects T cell activation and differentiation only at the onset of psoriasis, the effect of EKC on dendritic cells and keratinocytes was investigated.

**Figure 7:**
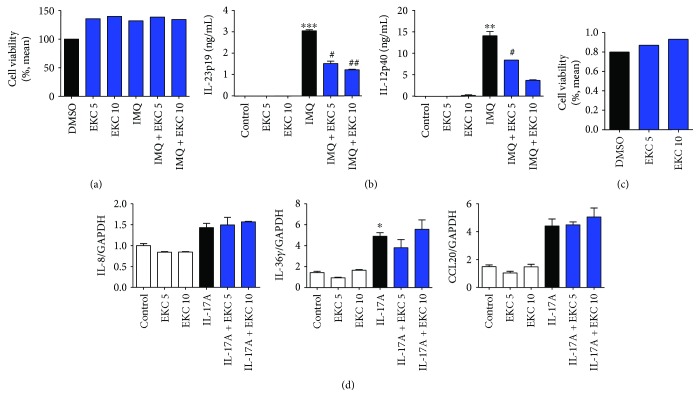
Reduced production of IL-12 and IL-23 in IMQ-stimulated dendritic cells by EKC. (a) JAWSII cells were plated at 2 × 10^5^ cells/well in a 96-well plate. EKC (5 *μ*g/mL and 10 *μ*g/mL) dissolved in DMSO (final concentration of 0.05%) was added to the cells, which were then cultured for 24 h with or without IMQ (1 *μ*g/mL). Cytotoxicity was analyzed by MTT assay. (b) JAWSII cells (1 × 10^6^) were plated in a 24-well multiplate, cultivated with EKC (5 *μ*g/mL and 10 *μ*g/mL), and treated with or without IMQ (1 *μ*g/mL). After 18 h, total RNA was isolated from each sample and reverse-transcribed cDNA was analyzed by quantitative real-time PCR (qPCR). Values represent the mean ± SD. ^∗∗^*p* < 0.01; ^∗∗∗^*p* < 0.001 compared with control (0.05% DMSO); ^#^*p* < 0.05; ^##^*p* < 0.01 compared with the IMQ group. (c) HaCaT cells were plated at 2 × 10^5^ cells/well in a 96-well plate. EKC (5 *μ*g/mL and 10 *μ*g/mL) dissolved in DMSO (final concentration of 0.05%) was added, and the cells were cultured for 24 h. Cytotoxicity was analyzed by MTT assay. (d) HaCaT cells (1 × 10^6^) were plated in a 24-well multiplate, cultivated with EKC (5 *μ*g/mL and 10 *μ*g/mL), and treated with or without human IL-17A (100 ng/mL). After 18 h, total RNA was isolated from each sample and reverse-transcribed cDNA was analyzed by quantitative real-time PCR (qPCR). Values represent the mean ± SD. ^∗^*p* < 0.05 compared with control (0.05% DMSO).

In IMQ-induced psoriasis model, dendritic cells are stimulated by TLR7/8 ligand (IMQ) and produce IL-12 and IL-23, which lead to accelerate Th1 and T17 cell differentiation, respectively [40]. Thus, JAWS II cells, murine dendritic cell lines, were stimulated by IMQ with or without EKC. The imiquimod had no cytotoxicity and was founded to be suitable for cell treatment (Figure 7(a)). The IMQ-stimulated cells significantly increased secretion of IL-12p40 and IL-23p19. The levels of these cytokines decreased in the EKC-treated cells (Figure 7(b)).

Additionally, aberrant activation and proliferation of the keratinocytes were caused by Th17 signature cytokines in psoriatic skin [41]. Under these conditions, keratinocytes produced cytokines and chemokines, such as IL-8, IL-36*γ*, and CCL20, which recruit inflammatory cells and exacerbate skin inflammation [42–44]. EKC did not affect the viability of the human keratinocyte HaCaT cell line (Figure 7(c)). Cells were challenged by IL-17A, with or without EKC, and total RNA was extracted. Although the levels of IL-8, IL-36*γ*, and CCL20 were increased by IL-17A (no significant changes except for IL-36*γ*), they were not modulated by EKC (Figure 7(d)).

Consequently, it was concluded that EKC affected the IMQ-induced activation of dendritic cells but not Th17-stimulated keratinocytes.

## 4. Discussion

*E. kansui,* an herb of the family of Euphorbiaceae, has been used to treat various diseases related to excessive inflammation [45, 46]. Previous studies have identified that *E. kansui* decreased the differentiation of Th17 cells and relieved inflammatory bowel disease, a Th17-associated autoimmune disease (unpublished data).

In this study, it was investigated whether psoriasis, a known autoimmune disease with pathogenesis focused on Th17 cells, was affected by *E. kansui.* The topical application of imiquimod activated plasmacytoid dendritic cells, triggered downstream Th1 and Th17 cell-mediated adaptive immunities, and resulted in mice with similar lesions to human psoriasis [26]. This quick and cost-effective model furthered the elucidation of pathogenic mechanisms and the evaluation of new treatments for psoriasis [47, 48].

Recent studies have suggested that regulatory B cells, *γδ* T cells (dendritic epidermal T cells [DETCs]), and innate immune cells perform an essential role in the pathogenesis of psoriasis and that regulation of these cells was also important [49–52]. However, the IL-17A/IL-23 axis was still emphasized as an important factor in the treatment of psoriasis [15, 26].

Treatment with the methylene chloride fraction of *E. kansui* radix (EKC) reduced IL-17A and IFN-*γ* significantly in Th17- and Th1-driving conditions. Although the in vitro data indicated that EKC increased TNF-*α* production, the clinical data from patients with psoriasis demonstrated a spectacular improvement with an IL-17 blockade rather than increased TNF [53]. Furthermore, recent studies have reported that TNF-alpha inhibitors therapy exacerbated psoriasis or induced new onset of psoriatic skin lesions in some cases [54, 55]. Thus, the curtailed symptoms in the psoriasis model may be attributed to the effective reduction of IL-17 by EKC; IL-17 is considered a major factor in the treatment of psoriasis.

The severity of inflammation (erythema, scaling, and thickness) was alleviated by EKC administration in a dose-dependent manner. The histological analysis demonstrated that acanthosis in the EKC group was more improved than that in the MTX group whereas the infiltration of inflammatory cells in the MTX group was similar to that observed after the highest dose of EKC administration. The relief of symptoms was associated with the suppression of Th17 and Th1 differentiation.

In the spleen, similar to a previous study, the percentage of T cell population (CD4^+^ and CD8^+^ T cells) was decreased by IMQ [26]. However, when considering the total number of splenocytes, the number of T cells did not change (data not shown). In addition, elevated percentages of splenic CD4^+^ IL-17A^+^ IFN-*γ*^−^ cells and CD8^+^ IFN-*γ*^+^ cells were observed in IMQ-treated mice. In contrast, CD4^+^ IFN-*γ*^+^ double positive cells were almost absent [26]. It was estimated that the reduction of IL-17A, IL-22, TNF-*α*, and ROR-*γ*t compared to that in the IMQ-treated group occurred through the inhibition of Th17 cells (CD4^+^ IL-17A^+^ IFN^−^ cells) by EKC and that the reduction of IFN occurred through the inhibition of CD8^+^ IFN^+^ cells. The *γδ* T cells in the skin-draining lymph node of psoriasis produce approximately 10 times more IL-17A and IL-22 than CD4 T cells [48, 56]. Also, these cells express the Th17-specific transcription factor, ROR*γ*t [57]. The reduction of cytokines and ROR*γ*t expression in the draining lymph node was considered to be related to the effect of EKC on *γδ* T cells. However, the effect of EKC on *γδ* T cells requires further study.

IL-23 and IL-12 are cytokines with critical roles in the differentiation and growth of Th17 and Th1 in psoriasis [58, 59]. IMQ increases the activation and population of dendritic cells and macrophages [60, 61]. EKC not only reduced dendritic cell populations in the spleen but also decreased IL-12 and IL-23 expression in the spleen and skin. Moreover, the in vitro experiments have shown that the activation of dendritic cells was suppressed by EKC. These results suggested that EKC inhibited dendritic cell activity independently of Th17 inhibition.

IMQ treatment was shown to increase serum IL-17A as well as skin inflammation, which led to systemic immune activation [62]. When EKC was administered, the level of IL-17A and IL-12p40 in both skin tissue and blood was decreased. Th17 cells induce expansion of cognate B cells, immunoglobulin class switching, and the increase in the number of B cells. Moreover, Th1 cells have a character promoting higher IgG2 whereas Th17 cells support higher IgG1 levels [63]. As the B cell population was increased in the spleen, blood IgG1 was investigated and the IgG level was found to be decreased in a dose-dependent manner by EKC treatment. In contrast, the number of B cells in splenocytes was not diminished by EKC administration. According to these results, the decrease in IgG1 seems to be related to the prevention of Th17-mediated class switching by inhibiting Th17 cells rather than directly involved in B cells.

In order to determine if the suppression of I*κ*B phosphorylation in skin resulted from a decrease in IL-17A or EKC affected keratinocyte activation, keratinocyte cell lines were activated by IL-17A, with or without EKC. The results elucidated that EKC did not affect keratinocytes. These results have drawn that suppression of EKC-induced Th17 resulted in relief of psoriasis through interruption of the phosphorylation of I*κ*B in skin.

In conclusion, this study revealed that reduced inflammation in the EKC-treated group was caused by the inhibition of Th17 but not keratinocytes. EKC suppressed not only the differentiation of Th17 but also the activation of dendritic cells. EKC is expected to be suitable for the treatment of patients with early and intermediate stages of psoriasis. Additionally, the effect of single compounds extracted from EKC should also be assessed as potential therapies for psoriasis.

## Figures and Tables

**Table 1:** Statistical analysis of the total score.

Day	Noninduction	IMQ + water	IMQ + MTX	IMQ + EKC low	IMQ + EKC mid	IMQ + EKC high
	Mean ± SD	Mean ± SD	Mean ± SD	Mean ± SD	Mean ± SD	Mean ± SD
1	2.00 ± 0.00	1.50 ± 0.58	2.00 ± 0.00	1.75 ± 0.50	2.25 ± 0.50	2.00 ± 0.82
3	2.33 ± 0.58^∗∗∗^	6.98 ± 0.60^###^	5.73 ± 0.90	5.88 ± 0.35^∗^	6.25 ± 0.45	5.20 ± 0.54^∗∗^
5	2.73 ± 1.22^∗∗^	8.75 ± 0.73^##^	6.70 ± 0.53^∗∗^	7.33 ± 1.18	6.80 ± 0.43^∗∗^	6.55 ± 0.73^∗∗^
7	3.00 ± 0.00^∗∗∗^	9.33 ± 0.50^###^	7.73 ± 0.42^∗∗^	7.95 ± 0.72^∗^	7.53 ± 0.76^∗^	7.40 ± 0.08^∗∗^

^∗^ *p* < 0.05; ^∗∗^*p* < 0.01; and ^∗∗∗^*p* < 0.001 compared with imiquimod + water group. ^##^*p* < 0.01; ^###^*p* < 0.001 compared with noninduction group.
